# Isolation of Halophilic and Halotolerant Bacterial Strains, Screening for Bioactive Compounds and Characterisation of Metabolites Produced by *Pseudoalteromonas* sp. ASV78

**DOI:** 10.1111/1758-2229.70159

**Published:** 2025-07-30

**Authors:** Maia Azpiazu‐Muniozguren, Elena Valgañón‐Pérez, Minerva García‐Martínez, Alba Rodriguez‐Paniagua, H. Poppy Clark, Carlos Justicia, Jesús Martín, Mercedes de la Cruz Moreno, Fernando Reyes, Lorena Laorden, Irati Martinez‐Malaxetxebarria, Ilargi Martinez‐Ballesteros

**Affiliations:** ^1^ MikroIker Research Group, Immunology, Microbiology and Parasitology Department, Faculty of Pharmacy University of the Basque Country UPV/EHU Vitoria‐Gasteiz Spain; ^2^ Bioaraba Microbiology, Infectious Diseases, Antimicrobial Agents, and Gene Therapy Vitoria‐Gasteiz Spain; ^3^ Department of Organic Chemistry I, Faculty of Pharmacy and Lascaray Research Center University of the Basque Country UPV/EHU Vitoria‐Gasteiz Spain; ^4^ Department of Chemistry, Marine Biodiscovery Centre University of Aberdeen Aberdeen UK; ^5^ Fundación MEDINA Centro de Excelencia en Investigación de Medicamentos Innovadores en Andalucía Armilla Granada Spain

**Keywords:** antimicrobial, biosynthetic gene clusters, halophilic bacteria, surface‐active compounds

## Abstract

Halophilic and halotolerant microorganisms are an important source of natural products. In this study, a collection of 150 bacterial isolates from a continental saltern (Añana Salt Valley, Spain) was obtained, identified and screened for the production of antimicrobial and/or surface‐active compounds. The identification of the isolates showed the predominance of the *Pseudomonadota* phylum with 106 isolates (70.7%) classified under this taxon. Subsequent bioassays identified 20 antimicrobial producers and 14 isolates with the ability to both reduce surface tension and emulsify. One of them, namely, ASV78 was characterised in detail. *Pseudoalteromonas* sp. ASV78 showed simultaneous production of pentabromopseudilin and bromophene, with antibacterial activity, as well as glycolipids with the ability to reduce surface tension and glycoproteins with effective emulsifying properties. The low concentration of pentabromopseudilin (MIC values ranging from 0.02 to 0.04 μg/mL) at which it is effective against *S. aureus* ATCC 29213 was noteworthy, as was the compound's activity against other clinically relevant Gram‐negative and Gram‐positive bacteria. The presence of biosynthetic gene clusters (BGCs) in the genome of strain ASV78 that are not included among the known BGCs suggests the possibility that they encode unknown molecules. The applicability of the compounds synthesised by *Pseudoalteromonas* sp. ASV78 and the potential of the isolate collection require further investigation.

## Graphical abstract

A collection of 150 bacterial isolates from a continental saltern dominated by *Pseudomonadota* revealed 20 antimicrobial producers and 14 isolates with emulsifying and surface tension reducing properties. The strain ASV78 produces pentabromopseudilin and bromophene, which are active against Gram‐positive pathogens, as well as a glycolipid‐type biosurfactant and a glycoprotein‐type bioemulsifier.

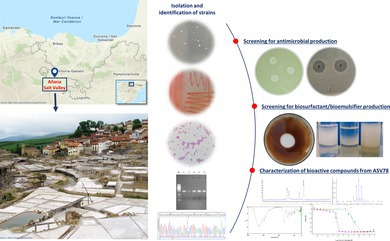

## 1 Introduction

Salinity is a distinctive environmental factor that continuously favours the metabolic adaptation and flexibility of halophilic and halotolerant microorganisms, allowing them to survive with minimal nutritional requirements. The genetic adaptation of these microorganisms to extreme solar radiation, ionic strength and desiccation makes them promising candidates for the discovery of new molecules (Raval et al. 2018). In particular, in order to address pressing health challenges (Leung et al. 2011), such as antibiotic resistance and contamination of soil or aquatic environments, focus has been placed on two classes of compounds of microbial origin: antimicrobials and surface‐active agents.

Bacterial resistance continues to emerge while the pace of antibiotic development slows. In this context, the biodiversity of halophiles has demonstrated significant potential for the production of new and unexplored antimicrobial agents (Thompson and Gilmore 2024). The use of halophilic biomolecules against drug‐resistant bacteria has attracted particular attention. One example is 13‐cis‐docosenamide, a unique antibacterial agent produced by *Vibrio* sp. against methicillin‐resistant *Staphylococcus aureus* (MRSA). In addition, the halophilic actinomycete *Nocardiopsis* sp. HR‐4, which was recovered from the soil of a salt lake in the Algerian Sahara, exhibits potent antimicrobial activity against drug‐resistant bacteria. This bacterium produces a novel natural product, 7‐deoxy‐8‐O‐methyltetrangomycin, which is also effective against MRSA (Santhaseelan et al. 2022).

The surface‐active compounds, whose uniqueness is attributable to the coexistence of a hydrophilic and a hydrophobic domain in the same molecule, include biosurfactants (low molecular weight compounds, which reduce the surface and/or interfacial tension between two immiscible phases) and bioemulsifiers (high molecular weight compounds, which allow the formation of oil‐in‐water or water‐in‐oil emulsions). These compounds have been shown to possess a broad spectrum of surface activities, including emulsification, dispersion, solubilisation, wetting and foaming (Perfumo et al. 2010). Consequently, their application in various fields is outstanding not only due to the aforementioned capabilities, but also because of their specific activity; high selectivity in a wide range of temperature, pH and salinity; biodegradability; and lower toxicity and environmental compatibility compared to synthetic surfactants (Gayathiri et al. 2022).

Despite the efforts made thus far, it is estimated that less than 10% of secondary metabolite gene clusters are expressed at levels that are sufficient for detection under current laboratory conditions. This has led to the development of genome‐based strategies for the discovery of new drugs and antibiotics. Evidence from the last decade suggests that pigmented species of the genera *Pseudoalteromonas*, among others, have the potential to produce a wide variety of bioactive compounds (specialised metabolites) of ecological and pharmaceutical importance, although most of these compounds remain uncharacterised (Chau et al. 2021; Nikolova and Gutierrez 2023).

It is evident that the current challenges require the study of innovative compounds with novel chemical structures, in conjunction with prospective areas of application. In this regard, in order to make the most of the opportunity to study the strains isolated from an active continental saltern located in the Añana Salt Valley in Álava, Spain (a hypersaline and unexplored environment) the following objectives were pursued in the present study: (i) to isolate and identify halophilic and halotolerant microorganisms from different water samples obtained from the saltern; (ii) to screen for antimicrobial and/or compounds with surface activity‐producing isolates; and (iii) to initiate the study of the compounds of interest.

## 2 Experimental Procedures

### 2.1 Water Sample Collection

Water samples were collected from the Añana Salt Valley (42°47′59.82″ N, 2°59′3.23″ W; altitude 574 m), located in western Álava, Basque Country, Spain at the same 12 sampling sites as described in Azpiazu‐Muniozguren et al. (2024). Samples from each site were stored in sterile, labelled glass bottles. All samples were then transported to the laboratory for further processing.

### 2.2 Isolation, Phenotype Description and Identification of Strains

In order to isolate halophilic and halotolerant prokaryotes, water samples were subjected to a series of filtration steps (Whatman Grade 113V, and Millipore 5 and 0.22 μm filters) and subsequently cultured on marine agar (MA; Conda) and MA supplemented with 23% NaCl (Sigma‐Aldrich) with each sample being cultured in duplicate. The media were then incubated at 25°C, with daily monitoring for colony formation over a period of up to 2 months. Subsequent subculturing of the isolates was performed on previously isolated medium until a pure culture was obtained. Pure cultures were maintained at −80°C in marine broth (MB; Conda) in 25% (v/v) glycerol. The morphological characteristics of all isolated colonies and the microscopic characteristics of the colony cells were examined.

For isolate identification, chromosomal DNA was extracted using the PrepMan Ultra Reactive (Applied Biosystems), according to the manufacturer's instructions. When this technique failed, the extraction was performed through the use of glass beads and boiling. DNA concentration was measured using a NanoDrop 2000 spectrophotometer (Thermo Scientific). Specific primers were used to amplify the 16S rRNA gene by PCR: 27F (5′‐AGAGTTTGATCMTGGCTCAG‐3′) and 1492R (5′‐GGTTACCTTGTTACGACTT‐3′) for bacteria (Goodfellow and Stackebrandt 1991), and 21F (5′‐TCCGGTTGATCCYGCCGG‐3′) and 1492R (5′‐GGTTACCTTGTTACGACTT‐3′) for archaea (DeLong 1992). In instances where no amplification was obtained, the Internal Transcribed Spacer (ITS) region of fungal ribosomal DNA was amplified using the universal primers ITS1 (5′‐TCCGTAGGTGAACCTGCGG‐3′) and ITS4 (5′TCCTCCGCTTA TTGATATGC‐3′) (White et al. 1990). The amplification was performed on a T100 thermal cycler (Bio‐Rad), using the appropriate amplification programme for each case. Sequencing of the purified PCR products was performed by the Sanger method (Stab Vida), and the resulting sequences were analysed using the Chromas 2.6.6 software (Technelysium) and CLUSTAL W webserver version 2.0.12 (Thompson et al. 1994). The identification of closely related species and their 16S rRNA gene sequence similarity was performed using the EzBioCloud website (www.ezbiocloud.net/identify). For isolates for which a complete or sufficiently long 16S rRNA gene sequence could not be obtained, a fragment similarity search was performed using the BLAST webserver (Altschul et al. 1990) for their approximate identification. A 16S rRNA gene‐based phylogenetic tree was constructed using the neighbour‐joining method in MEGA X software (Kumar et al. 2018) to establish the phylogenetic relationships between all the isolated strains.

### 2.3 Screening for Potential Biosurfactant/Bioemulsifier‐Producing Isolates

The entire collection was screened for biosurfactant/bioemulsifier production through a series of tests including Parafilm‐M test (Yalçın et al. 2018) and oil spreading test (Walter et al. 2010), for biosurfactant molecules production screening, and emulsification test (Willumsen and Karlson 1997; Walter et al. 2010), for bioemulsifiers production. Detailed information on the procedure for these tests can be found in the Supporting Information. MB supplemented with glucose (1% w/v) was used as a production medium (BPM). Falcon tubes containing 20 mL of BPM were inoculated with fresh cultures of the bacterial isolates and incubated at 25°C on a rotary shaker at 110 rpm. After 7 days, the cultures were centrifuged at 3100 *g* for 15 min at 4°C. The resulting cell‐free supernatants (CFS) were used directly in the screening assays. In all assays, sodium dodecyl sulphate (SDS) (1% w/v) was used as a positive control, while uninoculated BPM was used as a negative control. All assays had two replicates, except for the Parafilm‐M test, which had three replicates.

### 2.4 Screening for Potential Antimicrobial‐Producing Isolates

In order to determine the ability of the isolates to produce effective antimicrobial compounds, the entire collection was screened in an antagonism assay. Isolates that tested positive were then subjected to an agar diffusion assay (Flemer et al. 2012). Detailed information about these assays can be found in Supporting Information. The assays were performed against a number of clinically relevant microbial species, including *Escherichia coli* ATCC 25922, *Bacillus subtilis* CECT 356, *Staphylococcus aureus* ATCC 29213, *Pseudomonas aeruginosa* ATCC 27853, *Enterococcus faecalis* ATCC 29212 and *Candida albicans* ATCC 90029. The bacterial strains were maintained on Luria‐Bertani (LB; Scharlau) agar at 37°C. The fungal strain was maintained on Sabouraud dextrose agar (SDA; Scharlau) at 30°C. LB and SDA semi‐solid agar were used for the antagonism assay and Müller‐Hinton (MH; Scharlau) agar for the diffusion assay.

### 2.5 Characterisation of Isolate ASV78

Among all the isolates tested, the isolate ASV78 was selected for further analysis as it exhibited positive results for both surface‐active and antimicrobial activities. To optimise the production of the compounds of interest, MB medium supplemented with two carbon sources (1% w/v glycerol or glucose) was tested at two temperatures (25°C or 30°C) and two conditions (125 rpm or static) for a period of 5 days. Cell cultures were then centrifuged at 3100 *g* for 15 min at 4°C. The presence of activities of interest was then determined in accordance with the protocols outlined in Sections 2.3 and 2.4. The most favourable production conditions were established for all the subsequent analyses.

#### 2.5.1 Obtaining the Crude Extracts

In order to extract the antimicrobial compounds, the CFS was extracted twice with an equal volume of ethyl acetate, by means of vigorous shaking in a separating funnel. For the biosurfactant extraction, the CFS (pH 7.8) was adjusted to pH 2.0 with 1 M HCl prior to being extracted twice with an equal volume of ethyl acetate. The solvent of the organic phase in each case was then evaporated under vacuum using a Rotavapor R100 (Buchi, Switzerland) to obtain the crude extracts. Finally, to extract the bioemulsifier, the CFS was mixed with cold ethanol (1:1) and incubated overnight at −20°C. The bioemulsifier was then pelleted by centrifugation at 9000 rpm for 15 min at 4°C and dissolved in distilled water (1% w/v) before lyophilisation. The three crude extracts were weighed and stored at −20°C until further analysis. Each crude extract was tested for the presence of corresponding activities as described in the Supporting Information.

#### 2.5.2 Dereplication of Active Compounds by Liquid Chromatography‐Mass Spectrometry

Dereplication is defined as the analytical method used to rapidly identify already known natural products in samples from natural sources (Bradshaw et al. 2001). Dereplication of the active extracts of interest was performed using liquid chromatography high resolution mass spectrometry (LC–HRMS) in an Agilent 1200RR HPLC system coupled with a Bruker maXis Q‐TOF mass analyser, under analytical conditions previously described (Martín et al. 2014; Annang et al. 2015; Pérez‐Victoria et al. 2016).

#### 2.5.3 Purification and Characterisation of the Antimicrobial Compounds

A first fractionation of the extracted organic phase of the supernatant was performed on an automatic flash chromatography system (CombiFlash Rf, Teledyne Isco) using a linear gradient from 5% to 100% acetonitrile in water (in 35 min) with a final step of 100% acetonitrile (for 10 min), collecting 47 fractions. Fractions were concentrated to dryness in a centrifugal evaporator and fractions 27, 28 and 29 on the one hand and fractions 37, 38 and 39 on the other hand were pooled as they contained the two compounds of interest. These fractions were further separately purified by semi‐preparative reversed‐phase HPLC (XBridge Prep C_18_, 10 × 150 mm, 5 μm, 3.8 mL/min, UV detection at 210 and 280 nm, 1.8 mL/fraction) using a linear water‐acetonitrile gradient from 50% to 100% acetonitrile over 30 min to yield bromophene (0.5 mg, Rt 20 min) and pentabromopseudilin (0.9 mg, Rt 22 min). NMR spectra were recorded in CDCl_3_ on a Bruker Avance III spectrometer (500 and 125 MHz for ^1^H and ^13^C NMR, respectively) equipped with a 1.7 mm TCI MicroCryoProbe.

#### 2.5.4 Antibacterial Activity and Minimum Inhibitory Concentration of Purified Compounds

The antibacterial activity of the two purified compounds was tested using the broth microdilution method in a 96‐well plate format against *Staphylococcus aureus* ATCC 29213, *Pseudomonas aeruginosa* ATCC 27853, *Acinetobacter baumannii* ATCC 19606, *Enterococcus faecalis* ATCC 29212 and vancomycin‐resistant *Enterococcus faecium vanA* 15167 (clinical isolate) (Zhang et al. 2013; Vitorino et al. 2024). Vancomycin, ciprofloxacin and aztreonam were used as reference antibiotic compounds (positive controls). The initial assay concentration of the pure compounds was 80 μg/mL. To evaluate any synergistic activity, the initial assay concentration of each of the pure compounds was 40 μg/mL. Internal plate controls were included: blank control and growth control.

Turbidity (absorbance [Abs] at 600 nm) was measured at baseline (T0) and after 24 h of incubation (Tf) at 37°C, using an EnVision Microplate Reader (PerkinElmer). The minimum inhibitory concentration (MIC) was defined as the lowest concentration of the compound that inhibited ≥ 90% of the growth of a microorganism after overnight incubation. The Genedata Screener software, version 18.0.4‐Standard (Genedata Inc., Basel, Switzerland) was used to process and analyse the data. Three independent biological experiments (*n* = 3) were performed to determine the MIC values.

#### 2.5.5 Characterisation of the Extracts With Biosurfactant/Bioemulsifier Activity

The functional groups of the biosurfactant and bioemulsifier extracts were elucidated by infrared spectroscopic (IR) analysis. IR data spectra were obtained using a JASCO FT/IR‐4100 spectrometer equipped with a PIKE MIRacle single reflection ATR accessory (JASCO Corp., Tokyo, Japan). All measurements were performed at room temperature.

#### 2.5.6 Whole Genome Sequencing Analysis

The DNA extraction from ASV78 was performed using the NucleoSpin Tissue DNA Extraction Kit (Macherey‐Nagel), according to the manufacturer's protocol. Whole genome sequencing was performed on the Illumina NextSeq platform at the UPV/EHU Advanced Research Facilities (SGIker), combined with long sequencing on the MinION sequencer (Oxford Nanopore Technologies) at the University Hospital of Álava. After filtering for sequence quality, the raw Illumina reads and MinION long reads were de novo assembled using the Unicycler pipeline (Galaxy version 0.5.0+galaxy1). Genome‐based comparisons of strain ASV78 with its closest species were performed with average nucleotide identity (ANI) values computed using the OrthoANI Calculator tool available at EzBiocloud (www.ezbiocloud.net/tools/ani) (Yoon et al. 2017) and digital DNA–DNA hybridisation (dDDH) values computed using the genome‐to‐genome distance calculator (http://ggdc.dsmz.de/ggdc.php) (Meier‐Kolthoff and Göker 2019). A whole‐genome‐based taxonomic analysis was performed on the Type Strain Genome Server (https://tygs.dsmz.de) (Meier‐Kolthoff and Göker 2019) and visualisation was performed in iTOL v6 (Letunic and Bork 2024). Gene annotation was performed using the Rapid Annotation with Subsystems Technology (RAST) server v2.0 (Aziz et al. 2008) and Prokka v1.14.5 (Seemann 2014). The biosynthetic gene cluster (BGC) analysis was performed using antiSMASH v7.0 (Blin et al. 2023).

## 3 Results and Discussion

### 3.1 Isolation, Phenotype Description and Identification of Strains

A total of 150 halophilic and halotolerant isolates with different colony and cell morphologies were isolated from MA medium (89.3%, 134/150) and from MA containing 23% NaCl (10.7%, 16/150) (Table S1). Isolate identification at the domain level revealed an abundance of bacterial (97.3%) versus fungal (1.3%) and archaeal (1.3%) isolates. These results are reasonable considering the culture media used in this study, which are media for the cultivation of heterotrophic marine bacteria (Patrick 1978).

The *Pseudomonadota* phylum predominated with 106 isolates (70.7%), consistent with what has been described in Rambla Salada, where the *Pseudomonadota* phylum comprises 72.5% of the total isolates (Luque et al. 2014). The other isolates were divided into the phyla *Actinobacteria* (21 isolates, 14.0%), *Firmicutes* (12 isolates, 8.0%), *Bacteroidetes* (seven isolates, 4.7%), *Euryarchaeota* (two isolates, 1.3%) and *Dikarya* (two isolates, 1.3%). It has been reported that in hypersaline habitats, the most abundant groups of cultivable bacteria belong to the phyla *Bacteroidetes*, *Pseudomonadota* and *Firmicutes* (Rani et al. 2020). At the genus level, the isolates were identified as belonging to 48 different genera and 95 different species. The genus *Halomonas* was identified as the one with the highest number of isolates (36 isolates; 24.0%), followed by *Pseudoalteromonas* (15 isolates; 10.0%). This occurrence has also been previously documented in the aforementioned study in Rambla Salada (Luque et al. 2014). The remaining genera contained between one and eight cultured representatives. In particular, the most frequently isolated species were *Halomonas sabkhae* , *Halomonas taeanensis* and *Pseudoalteromonas neustonica*, with seven isolates each. Finally, differences were observed in the distribution of taxa according to the isolation medium, since the two archaea ( *Haloarchaeobius iranensis* and *Halohasta litorea*) and the bacterial isolates of the genera *Salimicrobium*, *Pontibacillus* and *Salicola* were only isolated in MA containing 23% NaCl.

The application of the EzBioCloud database to identify the isolates revealed that some isolates exhibited values lower than 98.7% in the 16S rRNA gene sequence similarity. These values are considered to be below the threshold proposed by Chun et al. to distinguish between the two species (Chun et al. 2018). This means that 37 of the 150 isolates obtained in this study may represent novel taxa. The phylogenetic relationship between the isolates is shown in Figure S1.

### 3.2 Screening for Potential Biosurfactant/Bioemulsifier‐Producing Isolates

**FIGURE 1:**
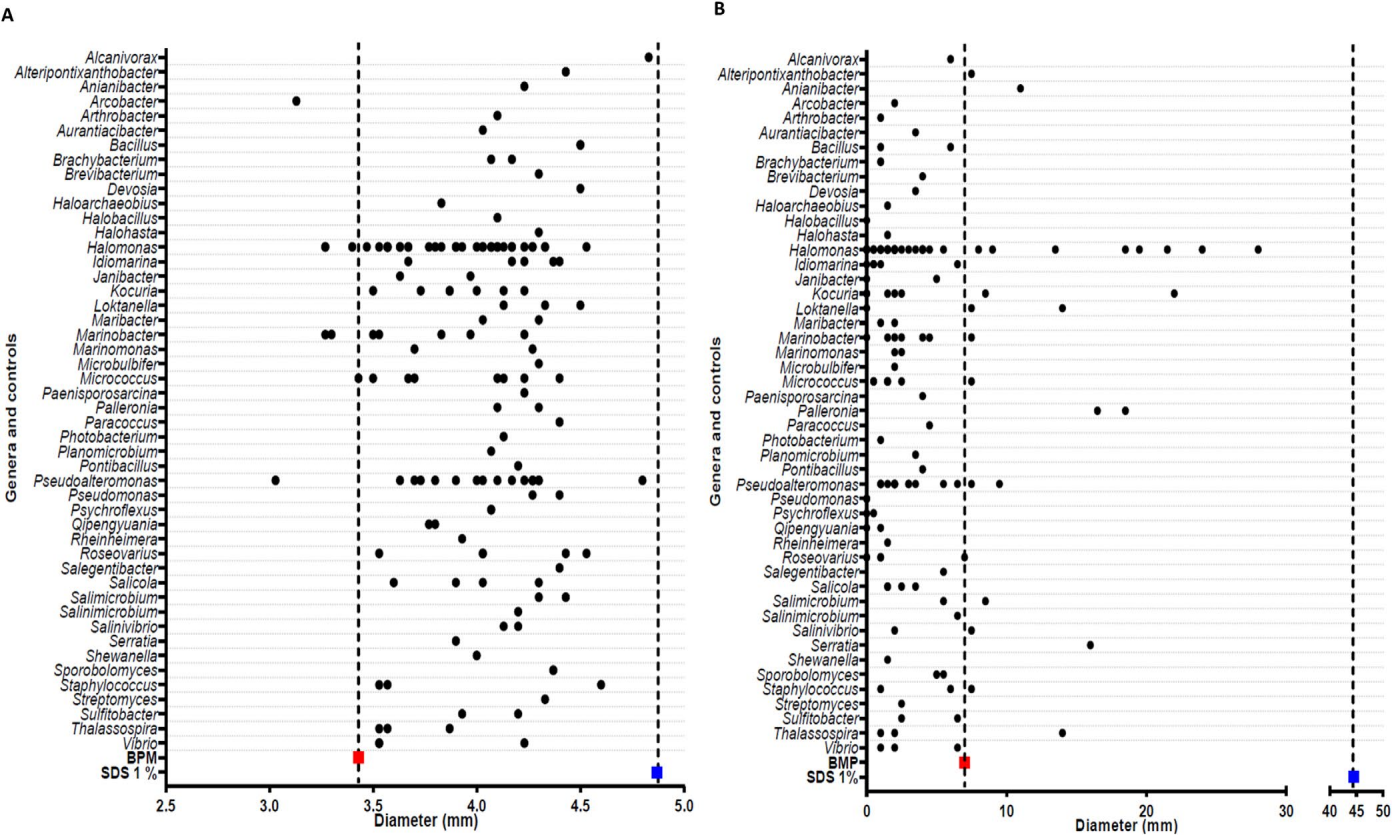
Results of the screening of isolates for the production of biosurfactants. Isolates are represented by dots, grouped by genus, showing the mean diameter of the droplet in (A) Parafilm‐M test, and the mean diameter of the clearing zone in (B) oil spreading test. The negative control (biosurfactant producing media, BPM) is shown in red and the positive control SDS (1% w/v) is shown in blue. Isolates with values higher than the negative control are considered positive for each test.

With the exception of two isolates that could not be recovered, the remaining 148 isolates were screened for their potential to produce biosurfactant/bioemulsifiers based on their ability to reduce surface tension by oil spreading and Parafilm‐M tests, and to emulsify oil and hydrocarbon compounds according to the emulsification index (EI_24_%). Illustrations of some of the results are presented in Figure S2, while the detailed results are summarised in Table S2. The use of these primary screening methods was consistent with similar studies (Youssef et al. 2004; Yalçın et al. 2018). The mean results obtained for each isolate in the Parafilm‐M test and the oil spreading test are shown in Figure 1, grouped by genus. Isolates were considered to have biosurfactant production if the drop diameter in the Parafilm‐M test and the clearing zone they produced in the oil spreading test were greater than those seen with the control BPM (≥ 3.4 and ≥ 7.0 mm, respectively). Similar cut‐off values have been utilised in other studies to test positive for biosurfactant production (Yalçın et al. 2018; Rani et al. 2020).

The results showed that 141 isolates (95.3%) were biosurfactant producers by the Parafilm‐M test. Among them, two isolates, ASV36T and ASV118, belonging to the genera *Alcanivorax* and *Pseudoalteromonas*, respectively, stood out because they resembled the positive control value. On the other hand, 26 isolates (17.6%) were classified as biosurfactant producers according to the oil spreading test. Two isolates of the genus *Halomonas*, ASV40 and ASV121, yielded the best results. All the isolates that were positive in the oil spreading test were also positive in the Parafilm‐M test. However, the large number of biosurfactant‐producing isolates detected by the Parafilm‐M test compared to those detected by the oil spreading test was striking, since both techniques are based on indirect measurement of the reduction in surface tension. This fact has been previously documented in other studies (Sun et al. 2018; Ghazi Faisal et al. 2023). The reason for this difference could be related to the fact that the reduction in surface tension does not always cause the oil to spread. This is because the decrease in surface tension might not be enough to overcome the forces that hold the oil together. This can lead to negative results in the oil spreading test. In addition, other factors such as the nature of the oil, the type of surfactant and the specific conditions of the system come into play for oil spreading (Lowast and Patel 2020; Zhang and Ducker 2023). Finally, results observed below the negative control (BPM) are indicative of an increase in surface tension, which is somehow related to changes in the fermentation process (e.g., changes in pH). This phenomenon has also been described for some strains in the study by Ziwei et al. (2023).

**FIGURE 2:**
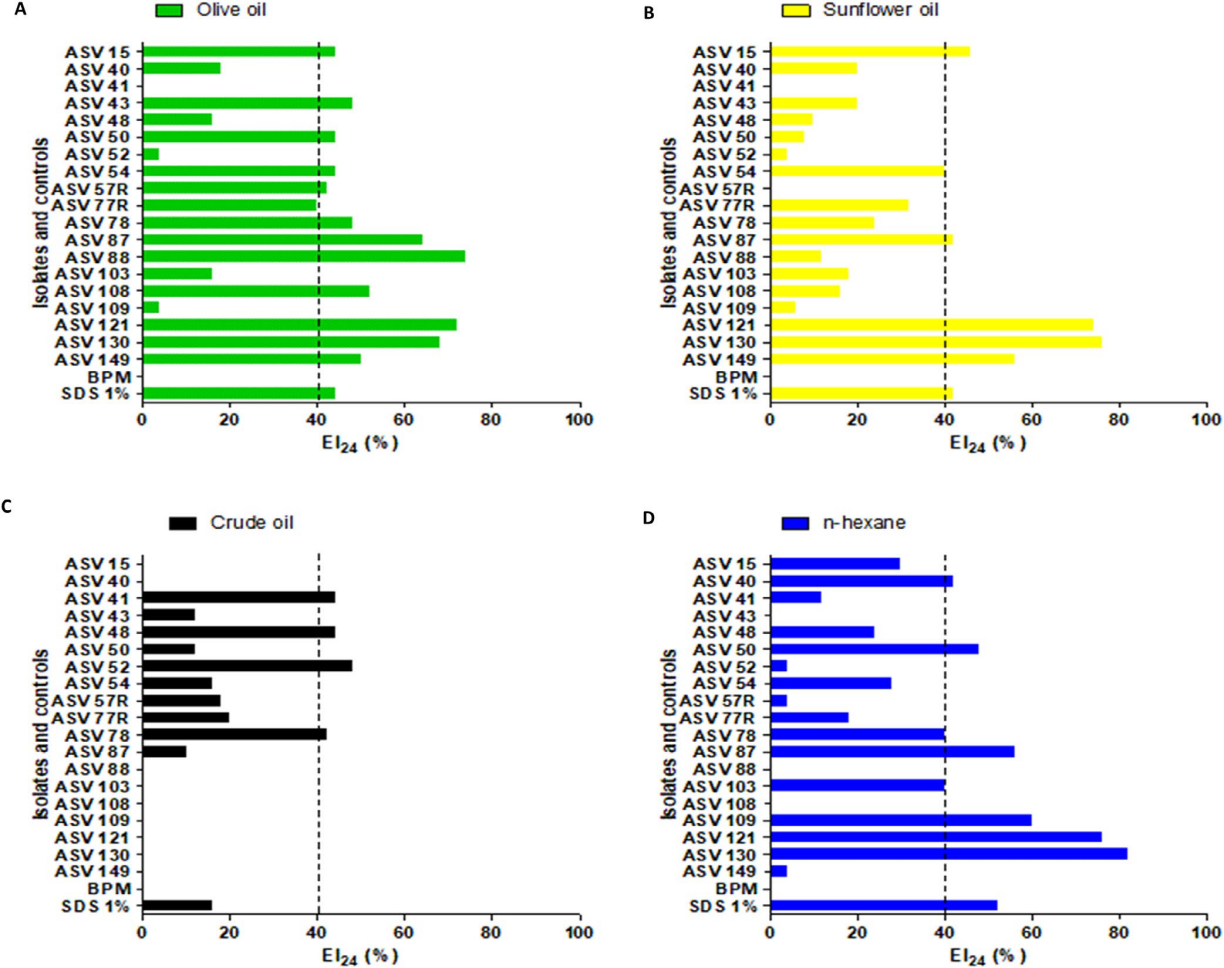
Emulsification indexes at 24 h interval (EI_24_) obtained by isolates with positive results with at least one of the hydrophobic substrates used in the study. (A) Olive oil; (B) sunflower oil; (C) crude oil; (D) n‐hexane. The results of the negative control (BPM) and the positive control SDS (1% w/v) results are also shown.

The isolates were subsequently screened for their ability to produce bioemulsifiers. A threshold of EI_24_ ≥ 40% was established as the criterion for identifying a promising bioemulsifier producers (Willumsen and Karlson 1997; Walter et al. 2010). Using this criterion, 19 isolates (12.8%) were identified as potential bioemulsifier producers (Figure 2). Among them, the bioemulsifiers with the highest EI_24_ were those produced by two isolates of the genus *Halomonas* (ASV121 and ASV130). On the other hand, only one strain (ASV78, *Pseudoalteromonas*) generated a stable emulsion with both hydrocarbons and olive oil.

In this study, 14 isolates (ASV40, ASV43, ASV50, ASV54, ASV57R, ASV77R, ASV78, ASV87, ASV88, ASV108, ASV109, ASV121, ASV130 and ASV149) were identified as both biosurfactant and bioemulsifier producers by testing positive in the three screening tests. Among them, the activity of some isolates belonging to the genera *Halomonas* and *Pseudoalteromonas* was outstanding. Interest in studying members of these genera has increased because they can produce biosurfactants and bioemulsifiers, making them promising candidates for bioremediation of contaminated sites (Rani et al. 2020; Nikolova and Gutierrez 2023). However, it is widely recognised that the culture medium plays an important role in the synthesis of biosurfactant/bioemulsifier compounds (Nurfarahin et al. 2018). This means that the isolates selected as biosurfactant or bioemulsifier producers in this study are worthy of further studies, as they may synthesise different surface‐active compounds from those currently known, depending on the growth conditions.

### 3.3 Screening for Potential Antimicrobial‐Producing Isolates

The isolates from the collection were subjected to a delayed antagonism assay. Twenty isolates showed a clear activity against at least one of the strains tested (Table 1). Among them, eight were active against both Gram‐positive and Gram‐negative bacterial strains, while antifungal activity was only observed in the isolate ASV55 in the well diffusion assay. On the other hand, the diffusion assay suggested that the antimicrobial compounds were produced by the third day of incubation and that degradation was evident after the 10th day. However, for some isolates, such activity was only observed in the delayed antagonism assay. This may indicate that the production of the antimicrobial compound was based on nutrient competition by the inhibitory strain (Moran et al. 2016). Illustrations of some of the results obtained in both antagonism and well diffusion assays are shown in Figure S3.

**TABLE 1:** Isolates that have demonstrated antimicrobial activity against at least one of the clinically relevant microbial species tested.

Isolate ID	Test strains																							
	*P. aeruginosa* ATCC 27853^T^				*E. coli* ATCC25922^T^				*B. subtillis* CECT 356^T^				*S. aureus* ATCC 29213^T^				*E. faecalis* ATCC 29212^T^				*C. albicans* ATCC 90029^T^			
	Zone of inhibition (mm)																							
	DAA	WDA			DAA	WDA			DAA	WDA			DAA	WDA			DAA	WDA			DAA	WDA		
		Day				Day				Day				Day				Day				Day		
		3	10	17		3	10	17		3	10	17		3	10	17		3	10	17		3	10	17
ASV1	8	−	−	−	−	−	−	−	−	−	−	−	−	−	−	−	−	−	−	−	−	−	−	−
ASV3	11	−	−	−	−	−	−	−	−	−	−	−	−	−	−	−	−	−	−	−	−	−	−	−
ASV7	20	−	−	−	−	−	−	−	12	−	−	−	25	−	−	−	−	−	−	−	−	−	−	−
ASV10	10	−	−	−	15	−	−	−	−	−	−	−	12	−	−	−	−	−	−	−	−	−	−	−
ASV11	10	−	−	−	−	−	−	−	−	−	−	−	−	−	−	−	−	−	−	−	−	−	−	−
ASV12	27	−	−	−	27	14	12	−	25	−	−	−	30	−	−	−	30	−	−	−	−	−	−	−
ASV13	−	−	−	−	9	15	11	−	−	−	−	−	−	−	−	−	31	−	−	−	−	−	−	−
ASV14	12	−	15	−	−	−	−	−	−	−	−	−	−	−	−	−	−	−	−	−	−	−	−	−
ASV25	12	−	12	−	15	−	−	−	16	−	−	−	10	−	−	−	13	−	−	−	−	−	−	−
ASV55	−	15	13	−	−	16	15	10	13	20	16	13	−	12	18	13	−	7	6	−	−	15	13	−
ASV78	12	−	−	−	−	−	−	−	20	−	−	−	17	−	−	−	−	−	−	−	−	−	−	−
ASV89	9	−	−	−	−	−	−	−	−	−	−	−	−	−	−	−	−	−	−	−	−	−	−	−
ASV106	−	−	−	−	−	−	−	−	21	18	−	−	−	−	−	−	−	−	−	−	−	−	−	−
ASV117	9	−	−	−	−	−	−	−	−	−	−	−	−	−	−	−	−	−	−	−	−	−	−	−
ASV118	9	−	−	−	−	−	−	−	−	−	−	−	−	−	−	−	−	−	−	−	−	−	−	−
ASV120	−	−	−	−	−	−	−	−	6	−	−	−	−	−	−	−	−	−	−	−	−	−	−	−
ASV121	−	−	−	−	−	−	−	−	6	−	−	−	−	−	−	−	−	−	−	−	−	−	−	−
ASV128	−	−	−	−	−	−	−	−	20	15	−	−	−	−	−	−	−	−	−	−	−	−	−	−
ASV129	−	−	−	−	−	−	−	−	19	17	−	−	−	−	−	−	−	−	−	−	−	−	−	−
ASV136	6	−	−	−	−	−	−	−	14	9	−	−	10	−	−	−	−	−	−	−	−	−	−	−

*Note:* −, negative. The diameter (mm) of the inhibition zone in the deferred antagonism assay (DAA) and well diffusion assay (WDA) is shown.

The major contributors to antibacterial activity in this study belonged to the genus *Pseudoalteromonas* (isolates ASV1, ASV3, ASV10, ASV11, ASV12, ASV13, ASV14, ASV25, ASV78, ASV89, ASV117, ASV118 and ASV136) (Table 1). Interestingly, all of them, except ASV13, inhibited the growth of *P. aeruginosa* . In addition to the possibility of nutrient competition inhibitory effect, another hypothesis for this occurrence is related to the ability of *Pseudoalteromonas* species to produce antibiofilm agents, such as alterocin, thereby suppressing the growth of *P. aeruginosa* , a recognised biofilm‐forming bacterium (Jouault et al. 2020). The remaining isolates with antimicrobial capacity belonged to the genera *Kocuria* (ASV106 and ASV128), *Halomonas* (ASV120 and ASV121), *Pseudomonas* (ASV55) and *Streptomyces* (ASV129) (Table 1), which have also been previously described as producing molecules with antimicrobial activity (Gardini et al. 2003; De Lima Procópio et al. 2012; Matthijs et al. 2014; Sadati et al. 2023). However, despite some reports of antifungal (Le and Yang 2018) and insecticidal (Du et al. 2020) activity of members of the genus *Salinivibrio*, their antibacterial capacity has not been described so far, which was seen in strain ASV7 in this study.

### 3.4 Further Study of ASV78 Isolate

#### 3.4.1 Obtaining the Crude Extracts

The culture conditions of *Pseudoalteromonas* sp. ASV78 that yielded the highest antibacterial and biosurfactant activities were used to prepare scaled‐up crude extracts. Thus, ASV78 was inoculated in MB medium (1000 mL) supplemented with glucose (1% w/v) and incubated at 25°C and 125 rpm for 5 days. A total of 700 mg of antimicrobial extract, 97 mg of biosurfactant extract and 350 mg of bioemulsifier extract were obtained following the procedures described in Section 2.5.1. The evaluation of the biological properties of each crude extract of ASV78 confirmed the antibacterial activity of the antimicrobial extract by TLC bioautography with two active bands against *S. aureus* ATCC 29213 (Figure S4). In addition, it was found that of the 700 mg of this crude extract, 1.4 mg belonged to the two identified antibacterial agents (specified later in the manuscript). The extracts of biosurfactant and bioemulsifier also yielded positive results. Indeed, a 1% solution of the crude biosurfactant extract demonstrated a droplet diameter of 5 mm in the Parafilm‐M test, while the crude bioemulsifier extract at 1% exhibited an EI_24_ of 57% with n‐hexane in the emulsification test. All these results indicated that *Pseudoalteromonas* sp. ASV78 synthesised biomolecules with different activities simultaneously under the same conditions.

#### 3.4.2 Characterisation of Antimicrobial Compounds

**FIGURE 3:**
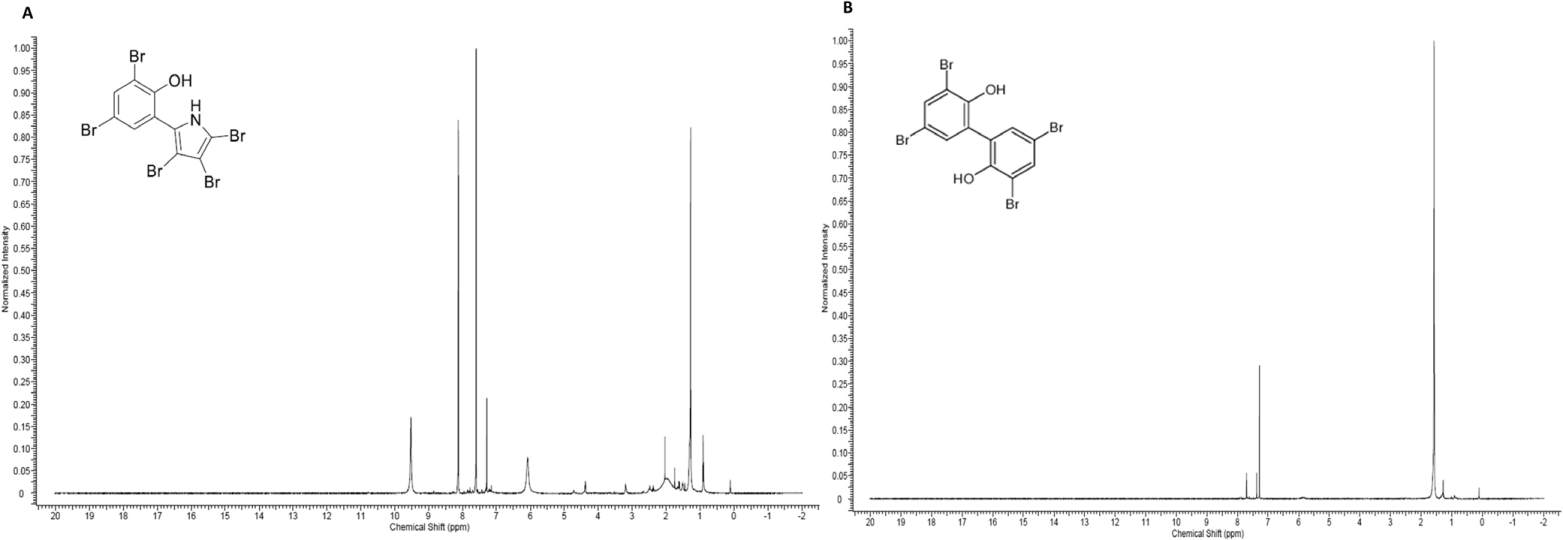
^1^H‐NMR spectra of (A) pentabromopseudilin and (B) bromophene, produced by *Pseudoalteromonas* spp. ASV78.

From the entire LC‐HRMS profile of the crude antimicrobial extract, 4 peaks were selected for subsequent analysis as they were clearly differential with respect to the fermentation medium (Figure S5). LC‐HRMS dereplication was performed in order to identify the components of the antimicrobial compound extraction. The dereplication process resulted in the identification of three peaks, which were identified as lumichrome (peak 4), pentabromopseudilin (peak 1) and bromophene (peak 2). The molecular formula obtained for peak 3 showed no coincidences in the Dictionary of Natural Products. Lumichrome was dereplicated as a match against the Fundación MEDINA library, matching the retention time, UV spectrum and molecular formula of the analysed peak with a standard stored in the spectral database (Figure S6). Lumichrome is a known degradation product of riboflavin, by photodegradation in neutral or acidic solutions and by enzymes in bacteria, with plant growth promoter activity (Phillips et al. 1999). Pentabromopseudilin was dereplicated by interpreting the molecular formula of a very well‐defined UV peak. The MS spectrum (Figure S7) showed a characteristic isotopic pattern with five bromine atoms, thus suggesting C_10_H_4_Br_5_NO as the molecular formula, with pentabromopseudilin being the unique match in the Dictionary of Natural Products. The compound was purified from a bioactive extract using reversed phase chromatography and preparative/semipreparative HPLC, and ^1^H‐NMR analysis (Figure 3A) confirmed its identity. Finally, the peak that was later identified as bromophene did not exhibit an interpretable (+)‐HRMS spectrum. Consequently, it was purified from the bioactive extract by reversed phase chromatography followed by preparative/semipreparative HPLC, in order to obtain a fraction that was suitable for NMR analysis. Negative mode LC‐LRMS produced a (−)‐MS spectrum that was interpreted, based on its isotopic distribution, to correspond to a molecular formula of C_12_H_6_Br_4_O_2_ with eight coincidences in the Dictionary of Natural Products. NMR analysis, including ^1^H (Figure 3B) and HSQC experiments, confirmed the presence of bromophene by comparison with literature data (Sundstrom et al. 2020). As described in this study, highly brominated metabolites from *Pseudoalteromonas* have also been documented. Specifically, pentabromopseudilin was previously isolated from *P. phenolica* and *P. luteoviolacea* and bromophene from *P. phenolica* (Fehér et al. 2010). However, this study describes the ability to simultaneously produce both compounds by the strain ASV78, for which the phylogenetically closest species is *Pseudoalteromonas neustonica* (16S rRNA gene similarity of 98.57%).

Despite the reported antibacterial activity of both identified compounds against methicillin‐resistant *S. aureus* , little is known about their broad spectrum (Fehér et al. 2010; Cascioferro et al. 2015). Moreover, the simultaneous production of both compounds may not be common. To the best of our knowledge, this has only been documented in a single strain, *Pseudoalteromonas* sp. CMMED 290 (Fehér et al. 2010), although the metabolic pathway is shared for the production of both compounds (Agarwal et al. 2014).

In this regard, the assay hold with the pure compounds demonstrated that both exhibited antibacterial activity against Gram‐positive bacteria, with pentabromopseudilin additionally displaying activity against Gram‐negative bacteria (Table 2). Specifically, pentabromopseudilin showed minimum inhibitory concentration (MIC) values ranging from 0.02 to 0.04 μg/mL against *S. aureus* ATCC 29213, and MIC values ranging from 10 to 20 μg/mL against *P. aeruginosa* ATCC 27853 (Table 2, left). Conversely, bromophene exhibited MIC values ranging from 2.5 to 5 μg/mL against *S. aureus* ATCC 29213 (Table 2, left).

**TABLE 2:** Antibacterial activity of pure compounds and controls.

Compounds	MIC (μg/mL)		Compounds	Activity (μg/mL)		
	*S. aureus* ATCC 29213	*P. aeruginosa* ATCC 27853		*A. baumannii* ATCC 19606	*E. faecalis* ATCC 29212	*E. faecium vanA* 15167
Pentabromopseudilin	0.02–0.04	10–20	Pentabromopseudilin	10–20	10–20	1.25–2.5
Bromophene	2.5–5	> 80	Bromophene	> 80	40–80	20–40
Synergy (1:1)	0.04–0.08	20–40	Synergy (1:1)	20–40	20–40	2.5–5
Vancomycin	0.5–1	ND	Vancomycin	ND	4–8	> 32
Ciprofloxacin	ND	0.25–0.5	Aztreonam	80–160	ND	ND

*Note:* MIC values on the right and antibacterial activity (MIC values obtained from one replicate) at the left.

Abbreviation: ND, not determined.

MIC values against *E. faecalis* ATCC 29212, vancomycin‐resistant *E. faecium vanA* 15167 and *A. baumannii* ATCC 19606 were determined using a single replicate assay. This was due to the scarcity of sample and therefore might require further validation in future studies. Pentabromopseudilin was found to inhibit the growth of *E. faecalis* ATCC 29212 at 10–20 μg/mL, vancomycin‐resistant *E. faecium* vanA 15167 at 1.25–2.5 μg/mL and *A. baumannii* ATCC 19606 at 10–20 μg/mL (Table 2, right). On the other hand, bromophene was observed to inhibit the growth of *E. faecalis* ATCC 29212 at 40–80 μg/mL and vancomycin‐resistant *E. faecium vanA* 15167 at 20–40 μg/mL (Table 2, right). No synergistic activity was observed between the two compounds against any of the clinically relevant microbial species that were tested.

The antibacterial activity of pentabromopseudilin against clinically relevant species such as *A. baumannii* , *E. faecium* and *S. aureus* is to be considered. The low concentration of pentabromopseudilin (MIC values ranging from 0.02 to 0.04 μg/mL) at which it is effective against *S. aureus* ATCC 29213 is surprising, especially when compared to the vancomycin concentrations (MIC values of 0.5–1 μg/mL in this study, and 0.52–0.59 μg/mL according to Lepe et al. (2014)). Therefore, it would be very interesting to assess the pentabromopseudilin spectrum against a more extensive array of pathogens and to further investigate its mechanism of action, which may be different from that of vancomycin, as evidenced by its activity against vancomycin‐resistant *E. faecium* and Gram‐negative bacteria.

#### 3.4.3 Characterisation of Surface‐Active Compounds

**FIGURE 4:**
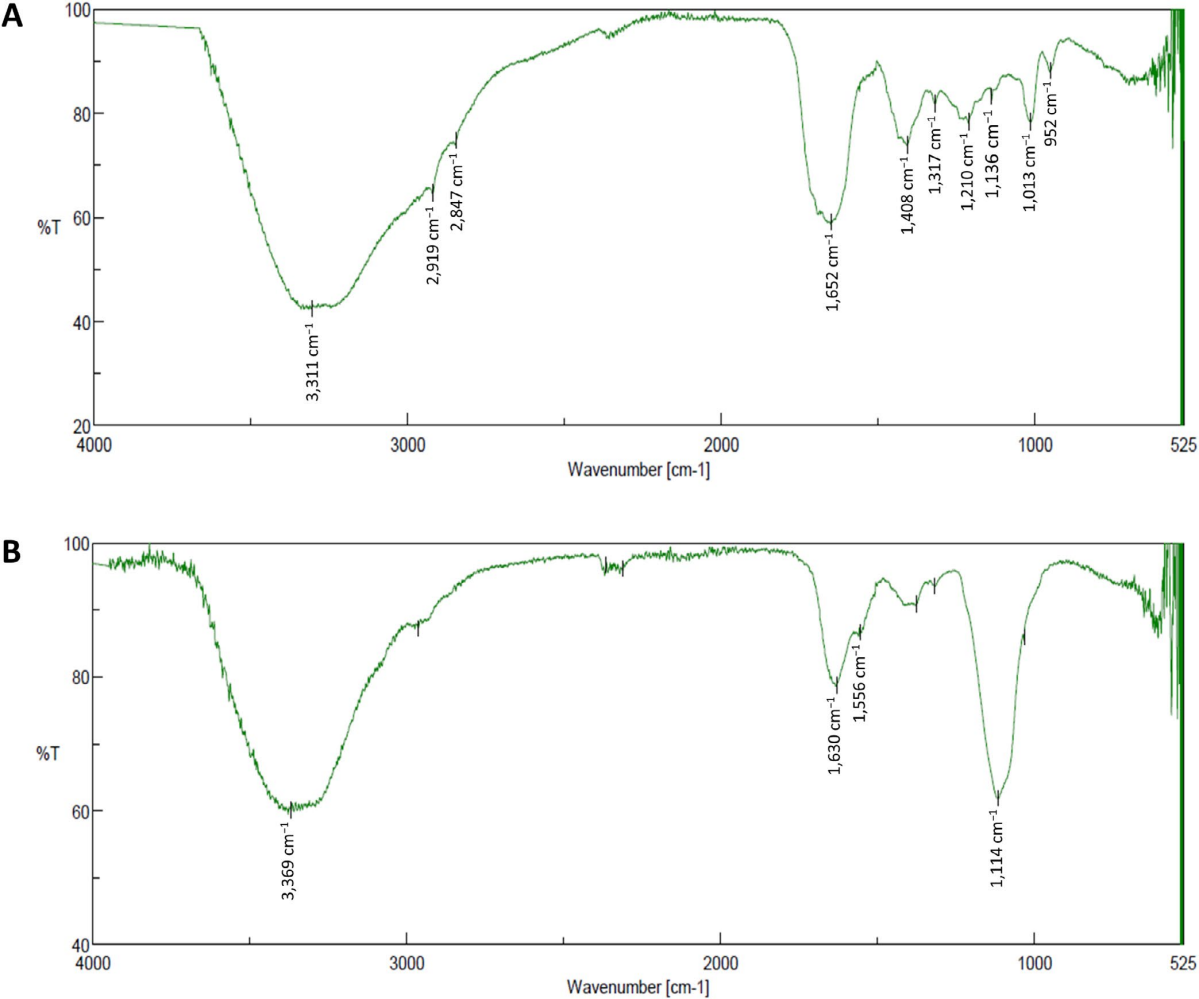
IR spectra of (A) biosurfactant extract and (B) bioemulsifier extract obtained from the fermentation broth of *Pseudoalteromonas* spp. ASV78.

IR spectroscopy was used to identify the chemical nature of the biosurfactant and bioemulsifier components of bioactive extracts in both assays. The IR spectrum of the biosurfactant extract revealed the presence of aliphatic hydrocarbon chains along with a polysaccharide moiety, thereby suggesting a glycolipid nature of the extract (Figure 4A). Absorption bands at 3311 cm^−1^ indicated the presence of —OH stretching of hydroxy groups. The presence of bands at 2919 and 2847 cm^−1^ corresponded to the C—H stretching mode of an aliphatic chain. The peak at 1652 cm^−1^ could be correlated with the presence of C=O groups. A peak at 1408 cm^−1^ indicated that the presence of CH bending vibrations of CH_3_ and CH_2_ may be attributed to polysaccharides. The peaks between 1317 and 1013 cm^−1^ indicated the presence of C—O stretching corresponding to the sugar moiety. The IR spectrum of the biosurfactant extract produced by *Pseudoalteromonas* sp. ASV78 was similar to those of other glycolipid biosurfactants reported in previous works (Thavasi et al. 2008; Singh and Tiwary 2016; Parthipan et al. 2018).

The IR spectrum of the bioemulsifier extract is shown in Figure 4B. Broad absorption bands indicating the presence of carboxylic groups (3369 cm^−1^, hydroxy stretching; 1630 cm^−1^, carboxyl C=O stretching; 1114 cm^−1^, carboxyl —C—O stretching) were observed. In addition, stretching vibrations were observed for the amide group at the wavelength of 1556 cm^−1^. They are associated with C—N and N—H groups, suggesting the presence of acylated amino sugars and proteins/peptides in the extract, indicating the presence of glycoproteins. The emulsifying activity of a glycoprotein exopolymer produced by *Pseudoalteromonas* spp. has been described previously (de la Maza et al. 1998; Gutierrez et al. 2008).

It is well established that surface‐active compounds are involved in the ‘pseudosolubilisation’ strategy, which aims to enhance bioavailability and facilitate access to hydrophobic compounds. Moreover, the genus *Pseudoalteromonas* is typical among hydrocarbon‐degrading microorganisms; therefore, it is not surprising that a significant proportion of biosurfactant or bioemulsifier producers are also found in this genus (Perfumo et al. 2010). Consequently, further characterisation and study of the potential applicability of the active compounds produced by *Pseudoalteromonas* sp. ASV78 could be promising.

#### 3.4.4 Whole Genome Sequencing

The ASV78 genome sequence was obtained using a combination of MinION and Illumina approaches yielding 19 contigs (the data is accessible at the NCBI under the BioProject accession number PRJNA1218939). The genome had a G + C DNA content of 41.5% and 5,215,181 bp. The assembled contigs were analysed using Prokka and a total of 4674 genes were identified. Among them, 4548 were identified as protein coding genes (CDSs). Nevertheless, merely 55.5% of the CDSs were assigned a function; the remainder were annotated as hypothetical protein CDSs. This underscores the need for further investigation into the functions of these genes.

According to the 16S rRNA gene sequence extracted from the genome (1547 bp), phylogenetically closely related type strains to ASV78 were *Pseudoalteromonas neustonica* (98.57%) and *Pseudoalteromonas prydzensis* (98.55%). However, the 16S rRNA gene is a poor marker of species phylogeny, at least in *Pseudoalteromonas*, due to the variability of the gene within individual genomes and the frequent occurrence of identical alleles in multiple genomes (Paulsen et al. 2020). Therefore, Overall Genome Relatedness Index (OGRI) analysis is highly recommended to classify species as known or novel species based on their genomic relatedness to type strains (Chun and Rainey 2014). Genomic analyses between ASV78, *P. neustonica* PAMC 28425 (ASM165313v1) and *P. prydzensis* ACAM 620 (ASM1492535v1) showed differences, since their genome size varied from 5.0 to 5.2 Mb and the G + C content varied from 39.5% to 41.5%. In addition, dDDH values were below the cut‐off value of 70% for the two type strains (between 23.9% and 39.1% similarity). Strain ASV78 was also compared based on average nucleotide identity (ANI), sharing the major identity with *P. prydzensis* (89.93%). However, the ‘gold standard’ for species identification using ANI is often accepted as 95%–96% (Chun et al. 2018). This means that because the ANI value is less than 95%, ASV78 does not belong to any known species in the genus. Visualisation of the phylogenomic tree of *Pseudoalteromonas* species based on genome distances showed the strain ASV78 in a single branch separated from the rest of the species (Figure S8). Although all genomic approaches indicated that ASV78 may be a novel species, further phenotypic analyses are needed. Genome sequence analysis with antiSMASH revealed a total of seven regions putatively encoding BGCs (Table S3). The total length of the predicted BGCs is approximately 250 Kb, representing ~4.8% of the ASV78 genome. The number of predicted BGCs relative to genome size is in the average range found in prokaryotes (Cimermancic et al. 2014). One of the predicted BGCs showed 100% similarity to the known BGC pentabromopseudilin. In this regard, Agarwal et al. emphasised that the bpm biosynthetic gene cluster is responsible for the production of a limited number of polybrominated diphenyl ether (PBDE) compounds, including bromophene and pentabromopseudilin (Agarwal et al. 2014). In this sense, our results are consistent with the confirmed presence of both compounds in the extract via LC–HRMS. However, a phylogeny of the genus revealed that numerous clades do not contain these genes (Busch et al. 2019). In fact, the detection of the bioactive compounds was mostly restricted to the clades containing the *P. luteoviolacea* and *P. phenolica* type strains (Busch et al. 2019), and not to *P. neustonica* as was the case for strain ASV78.

Two other BGSs showed 75% and 45% similarity with the desferrioxamine E BGC (Barona‐Gómez et al. 2004) and APE Vf BGC (Cimermancic et al. 2014), respectively. Desferrioxamine E and its analogues are siderophores widely produced by *Streptomyces* and related bacteria (Yamanaka et al. 2005). However, in the *Pseudoalteromonas* genera where desferrioxamine is found (such as *P. spongiae*, *P. ruthenica*, *P. piratica* and *P. tunicata*), it is desferrioxamine B (Paulsen et al. 2019). Finally, aryl polyenes are yellow pigments widespread among Gram‐negative pathogens, such as APE_Ec_ from uropathogenic *E. coli* (Johnston et al. 2021). They are structurally and functionally related to carotenoids and confer protection against photo‐oxidative damage and lipid peroxidation (Schöner et al. 2016). A yellowish colony colour was observed when ASV78 was cultured on agar plates and is probably related to its genomic ability to synthesise aryl polyenes. The rest of the BGCs showed low (8% with N‐myristoyl‐d‐asparagine) or no similarity to known BGCs, suggesting the potential to encode novel molecules. In this sense, among the unidentified BGCs could be biosurfactants produced by strain ASV78, since in other studies non‐ribosomal peptide synthetases (NRPSs) have been identified as surfactins or fengycin (Ding et al. 2024), which are recognised biosurfactants.

## 4 Conclusion

Extreme environments such as continental salterns are a source of new and diverse microorganisms able to biosynthesise biomolecules of great utility for both current and future biotechnological challenges. The biosynthesis of antimicrobial and surfactant compounds is a common feature between halophilic and halotolerant bacteria. However, the simultaneous production of both groups of compounds is less frequent. In the case of strain ASV78, its antibacterial activity has been attributed to the production of two brominated compounds, namely pentabromopseudilin and bromophene. In addition, the ability to synthesise glycolipid‐type biosurfactants and glycoprotein‐type bioemulsifiers has been confirmed by IR studies. Further investigation is needed to determine the applicability of the compounds synthesised by *Pseudoalteromonas* sp. ASV78 and the potential of the whole strain collection.

## Author Contributions

**Maia Azpiazu‐Muniozguren:** data curation, formal analysis, methodology, writing – original draft, writing – review and editing. **Elena Valgañón‐Pérez:** formal analysis, writing – review and editing. **Minerva García‐Martínez:** formal analysis, writing – review and editing. **Alba Rodriguez‐Paniagua:** formal analysis, writing – review and editing. **H. Poppy Clark:** formal analysis, writing – review and editing. **Carlos Justicia:** formal analysis, writing – review and editing. **Jesús Martín:** formal analysis, writing – review and editing. **Mercedes de la Cruz Moreno:** formal analysis, writing – review and editing. **Fernando Reyes:** methodology, formal analysis, validation, writing – review and editing. **Lorena Laorden:** methodology, resources, writing – review and editing. **Irati Martinez‐Malaxetxebarria:** funding acquisition, methodology, resources, writing – review and editing. **Ilargi Martinez‐Ballesteros:** conceptualization, funding acquisition, methodology, resources, supervision, writing – review and editing.

## Conflicts of Interest

The authors declare no conflicts of interest.

## Supporting information


**Figure S1.** Neighbour‐joining phylogenetic tree from 16S rRNA gene sequences showing the relationships between all the isolates and a reference sequence per genus. Isolates showing values below 98.7% for 16S rRNA gene sequence similarity are named with the genus followed by sp. *Caldithrix abyssi* was used as an outgroup. Bar, 0.20 substitutions per nucleotide position.
**Figure S2.** Visual example of some of the strains that produce surface‐active compounds in different assays compared to the negative control.
**Figure S3.** Visual example of some of the strains that produce antimicrobial compounds in different assays.
**Figure S4.** TLC‐based bioauthography of crude extract of ASV78 exhibiting antibacterial activity against *S. aureus* ATCC 29213.
**Figure S5.** The entire LC‐HRMS profile of the crude antimicrobial extract showing the four differential peaks with respect to the fermentation medium (without inoculum). (1) Pentabromopseudilin; (2) bromophene; (3) molecular formula without coincidences in the Dictionary of Natural Products; (4) lumichrome.
**Figure S6.** UV and (+)‐HRMS spectra of peak dereplicated as lumichrome. Ion at m/z 243.085 was interpreted as [M+H]+ of a compound with a molecular formula C_12_H_10_N_4_O_2_. Its UV spectrum and retention time of elution in standardised MEDINA HPLC system were in perfect concordance with lumichrome’s analytical data stored in MEDINA’s spectral library.
**Figure S7.** (+)‐HRMS spectrum of component dereplicated as pentabromopseudilin (lower) and simulated pattern of [C_10_H_5_Br_5_NO]+ (upper). Both spectra are perfectly compatible by isotopic pattern and exact masses of ions, allowing the assignment of the interpreted molecular formula to the analysed peak. Only pentabromopseudilin is described with the molecular formula C_10_H_4_Br_5_NO in the Dictionary of Natural Products.
**Figure S8.** Phylogenomic tree showing the position of the strain ASV78 and related strains of Pseudoalteromonas species based on genome distances as provided by the TYGS platform. Bootstrap values greater than 50% in the clade nodes are shown. Bar, 0.01 substitutions per nucleotide position.

**Table S1.** Halophilic and halotolerant isolates obtained from sampling sites. The isolate ID, culture medium from which they were isolated, macroscopic description of colony and cell morphology and Gram staining and identification based on 16S rRNA gene sequencing (closest species, strains and sequence similarity) are detailed.

**Table S2.** The raw data, means and standard deviations of the Parafilm‐M test, oil spreading test and emulsification test collected to analyse the production of biosurfactants according to the isolates.

## Data Availability

The data that supports the findings of this study are available in the Supporting Information of this article.
