# Supplementary material for: Isolation of Halophilic and Halotolerant Bacterial Strains, Screening for Bioactive Compounds and Characterisation of Metabolites Produced by Pseudoalteromonas sp. ASV78
Source: Environ Microbiol Rep. 2025 Jul 30;17(4):e70159. doi: 10.1111/1758-2229.70159 (PMC12308153; doi:10.1111/1758-2229.70159)
Supplement: Supplementary file 1 — Figure S1. Neighbour‐joining phylogenetic tree from 16S rRNA gene sequences showing the relationships between all the isolates and a reference sequence per genus. Isolates showing values below 98.7% for 16S rRNA gene sequence similarity are named with the genus followed by sp. Caldithrix abyssi was used as an outgroup. Bar, 0.20 substitutions per nucleotide position. Figure S2. Visual example of some of the strains that produce surface‐active compounds in different assays compared to the negative control. Figure S3. Visual example of some of the strains that produce antimicrobial compounds in different assays. Figure S4. TLC‐based bioauthography of crude extract of ASV78 exhibiting antibacterial activity against S. aureus ATCC 29213. Figure S5. The entire LC‐HRMS profile of the crude antimicrobial extract showing the four differential peaks with respect to the fermentation medium (without inoculum). (1) Pentabromopseudilin; (2) bromophene; (3) molecular formula without coincidences in the Dictionary of Natural Products; (4) lumichrome. Figure S6. UV and (+)‐HRMS spectra of peak dereplicated as lumichrome. Ion at m/z 243.085 was interpreted as [M+H]+ of a compound with a molecular formula C12H10N4O2. Its UV spectrum and retention time of elution in standardised MEDINA HPLC system were in perfect concordance with lumichrome’s analytical data stored in MEDINA’s spectral library. Figure S7. (+)‐HRMS spectrum of component dereplicated as pentabromopseudilin (lower) and simulated pattern of [C10H5Br5NO]+ (upper). Both spectra are perfectly compatible by isotopic pattern and exact masses of ions, allowing the assignment of the interpreted molecular formula to the analysed peak. Only pentabromopseudilin is described with the molecular formula C10H4Br5NO in the Dictionary of Natural Products. Figure S8. Phylogenomic tree showing the position of the strain ASV78 and related strains of Pseudoalteromonas species based on genome distances as provided by the TYGS platform. Bootst [file EMI4-17-e70159-s002.pdf]

Supplementary material

Environmental Microbiology Reports

Isolation of halophilic and halotolerant bacterial strains, screening for bioactive compounds and characterization of metabolites produced by *Pseudoalteromonas* sp. ASV78

Maia Azpiazu-Muniozguren[a,e], Elena Valgañón-Pérez[a], Minerva García-Martínez[a], Alba Rodriguez-Paniagua[b], H. Poppy Clark[c], Carlos Justicia[d], Jesús Martín[d], Mercedes de la Cruz Moreno[d], Fernando Reyes[d], Lorena Laorden[a,e], Irati Martinez-Malaxetxebarria[a,e], Ilargi Martinez-Ballesteros[a,e]*

[a]*MikroIker Research Group, Immunology, Microbiology and Parasitology Department, Faculty of Pharmacy, University of the Basque Country UPV/EHU, Paseo de la Universidad 7, 01006, Vitoria-Gasteiz, Spain.* maia.azpiazu@ehu.eus; evalganon003@ikasle.ehu.eus; minergarcia17@gmail.com; lorena.laorden@ehu.eus; irati.martinez@ehu.eus; ilargi.martinez@ehu.eus

[b]*Department of Organic Chemistry I, Faculty of Pharmacy and Lascaray Research Center, University of the Basque Country UPV/EHU, Paseo de la Universidad 7, 01006, Vitoria-Gasteiz, Spain.* alba.rodriguezp@ehu.eus

[c]*Marine Biodiscovery Centre, Department of Chemistry, University of Aberdeen, Aberdeen AB24 3UE, UK.* h.clark.21@abdn.ac.uk

[d]*Fundación MEDINA, Centro de Excelencia en Investigación de Medicamentos Innovadores en Andalucía, Avda. del Conocimiento 34, 18016 Armilla Granada, Spain.* carlos.justicia@medinaandalucia.es; jesus.martin@medinaandalucia.es; mercedes.delacruz@medinaandalucia.es; fernando.reyes@medinaandalucia.es

[e]*Bioaraba, Microbiology, Infectious Diseases, Antimicrobial Agents, and Gene Therapy, 01006, Vitoria-Gasteiz, Spain.* maia.azpiazu@ehu.eus; lorena.laorden@ehu.eus; irati.martinez@ehu.eus; ilargi.martinez@ehu.eus

*Corresponding author: ilargi.martinez@ehu.eus (I. Martinez-Ballesteros). MikroIker Research Group, Immunology, Microbiology and Parasitology Department, Faculty of Pharmacy, University of the Basque Country UPV/EHU, Paseo de la Universidad 7, 01006, Vitoria-Gasteiz, Spain. Tel.: +34 945013288; ORCID: 0000-0002-8867-1487

EXPERIMENTAL PROCEDURES

Screening for potential biosurfactant/bioemulsifier-producing isolates

Parafilm-M test

Twenty µL of each cell-free supernatant (CFS) was dropped onto a strip of Parafilm (hydrophobic surface) at room temperature. The drops were allowed to rest for 1 minute, and the diameter of the drop was measured using a magnifying glass and an eyepiece with a micrometer ruler (Yalçın *et al.*, 2018).

Oil spreading test

The oil spreading test was performed in glass Petri dishes (100 mm × 15 mm) containing a drop (20 µL) of crude oil, which was carefully layered over 20 mL of distilled water. Then, 10 µL of the CFS was carefully pipetted into the center of the oil layer. The diameter of the clear zone on the surface of the oil layer was measured (Walter *et al.*, 2010).

Emulsification index test

Emulsifying activity was determined by calculating the emulsification index (EI_{24}). Briefly, 2 mL of CFS was added to an equal volume of hydrophobic substrate (including olive oil, sunflower oil, crude oil and n-hexane). The mixture was vortexed at maximum speed for 2 minutes and allowed to stand for 24h at room temperature (Willumsen and Karlson, 1997; Walter *et al.*, 2010). The emulsification index after 24 h was quantified using the following equation:

$$\%\,EI_{24} = (Height\ of\ the\ emulsion\ layer/Total\ height)\ * 100$$

Screening for potential antimicrobial-producing isolates

Deferred antagonism assay

The antagonism assay was performed in duplicate according to the protocol described by Flemer et al. (Flemer *et al.*, 2012) with minor modifications. Bacterial cultures were adjusted to 10^8 cells/mL and 10 µL were spotted onto marine agar (MA) plates and incubated at 25 °C until the culture was 0.5-1 cm in diameter (for most isolates, 1-3 days of incubation was sufficient). These plates were then overlaid with 10 mL of Luria-Bertani (LB) soft agar inoculated with 10^7 cells/mL of each bacterial or fungal pathogen test strain. After solidification, the plates were incubated for 24 hours at 30 °C for the fungal strain and 37 °C for the bacterial strains. A zone of clearance in the overlaid agar indicated the production of an antimicrobial compound by the isolate of the collection.

Well diffusion assay

Isolates showing clear activity in the delayed antagonism assay were also tested in the well diffusion assay as described by Flemer et al. (Flemer *et al.*, 2012), with minor modifications. Isolates were inoculated into 20 mL of marine broth (MB) and incubated for 17 days at 25 ˚C with constant shaking (at 110 rpm). On days 3, 10 and 17, 1 mL of each culture was centrifuged at 11,000 rpm for 10 minutes to obtain a CFS. In parallel, Müller-Hinton agar plates were inoculated with 100 µL of the test pathogen at a concentration of 10^7 cells/mL after which holes were made in the agar using a sterile glass pipette (5 mm diameter). The wells were then filled with 40 µL of the CFS or the negative control (MB). Plates were incubated for 24-48 hours at 30 °C for the fungal strain and 37 °C for the bacterial strains. Growth inhibition around the punched wells predicted the presence of compounds with antimicrobial activity in the supernatant.

Characterizing isolate ASV78

Testing of the activity of the crude extracts

Each crude extract was tested for the presence of corresponding activities. To verify the presence of biosurfactant and bioemulsifier compounds in the corresponding extract, the crude extracts were tested in the same manner as described in Section 2.3 of the manuscript.

In addition, contact bioautography analysis was used to determine which of the bands separated on the thin layer chromatography (TLC) plate (Merck, Darmstadt, Germany) possesses antibacterial activity. For this purpose, the plate was placed on MH agar inoculated with the test pathogen at a concentration of 0.5 McFarland and it was left for 30 minutes to allow the compounds to diffuse from the plate to the agar medium. After incubation at 37 °C for 24 hours, the presence of a growth inhibition zone in the medium was observed indicating the band with antimicrobial activity (Chanthasena *et al.*, 2022).

References

Chanthasena, P., Hua, Y., Rosyidah, A., Pathom-Aree, W., Limphirat, W., and Nantapong, N. (2022) Isolation and Identification of Bioactive Compounds from *Streptomyces actinomycinicus* PJ85 and Their In Vitro Antimicrobial Activities against Methicillin-Resistant *Staphylococcus aureus*. *Antibiotics (Basel)* **11**: 1797.

Flemer, B., Kennedy, J., Margassery, L.M., Morrissey, J.P., O'Gara, F., and Dobson, A.D.W. (2012) Diversity and antimicrobial activities of microbes from two Irish marine sponges, Suberites carnosus and Leucosolenia sp. *Journal of applied microbiology* **112**: 289–301.

Walter, V., Syldatk, C., and Hausmann, R. (2010) Screening Concepts for the Isolation of Biosurfactant Producing Microorganisms. In *Biosurfactants*. Advances in Experimental Medicine and Biology. Sen, R. (ed). New York, NY: Springer New York, pp. 1–13.

Willumsen, P.A. and Karlson, U. (1997) Screening of bacteria, isolated from PAH-contaminated soils, for production of biosurfactants and bioemulsifiers. *Biodegradation* **7**: 415–423.

Yalçın, H.T., Ergin-Tepebaşı, G., and Uyar, E. (2018) Isolation and molecular characterization of biosurfactant producing yeasts from the soil samples contaminated with petroleum derivatives. *J Basic Microbiol* **58**: 782–792.

FIGURES

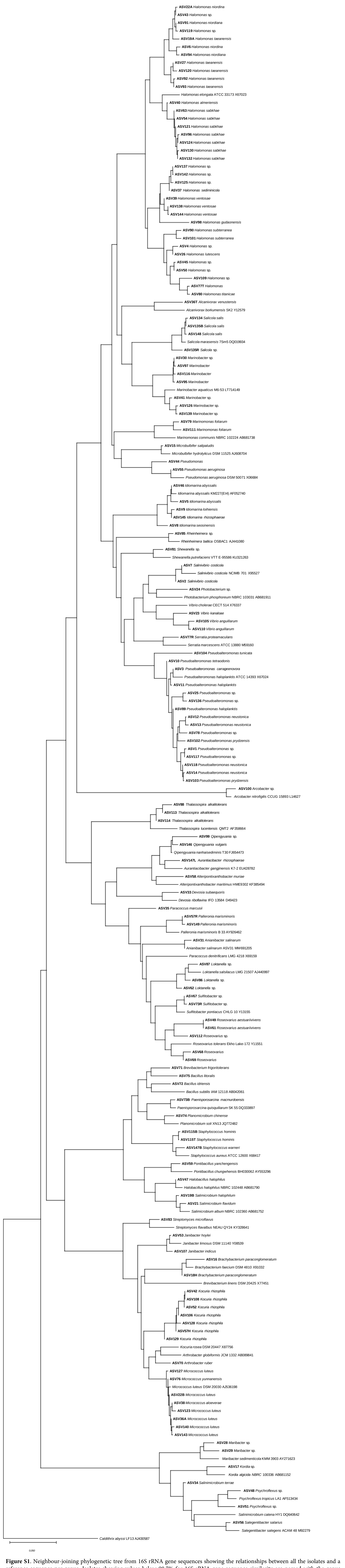

Figure S1. Neighbour-joining phylogenetic tree from 16S rRNA gene sequences showing the relationships between all the isolates and a reference sequence per genus. Isolates showing values below 98.7% for 16S rRNA gene sequence similarity are named with the genus followed by sp.. *Caldithrix abyssi* was used as an outgroup. Bar, 0.20 substitutions per nucleotide position.

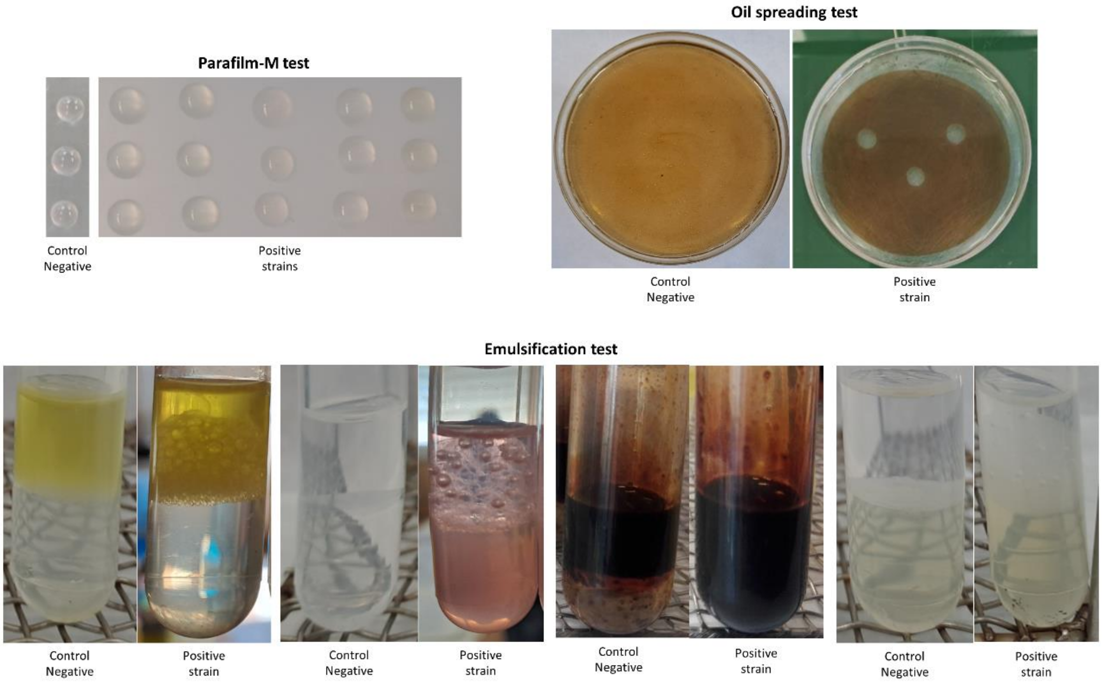

Figure S2. Visual example of some of the strains that produce surface-active compounds in different assays compared to the negative control.

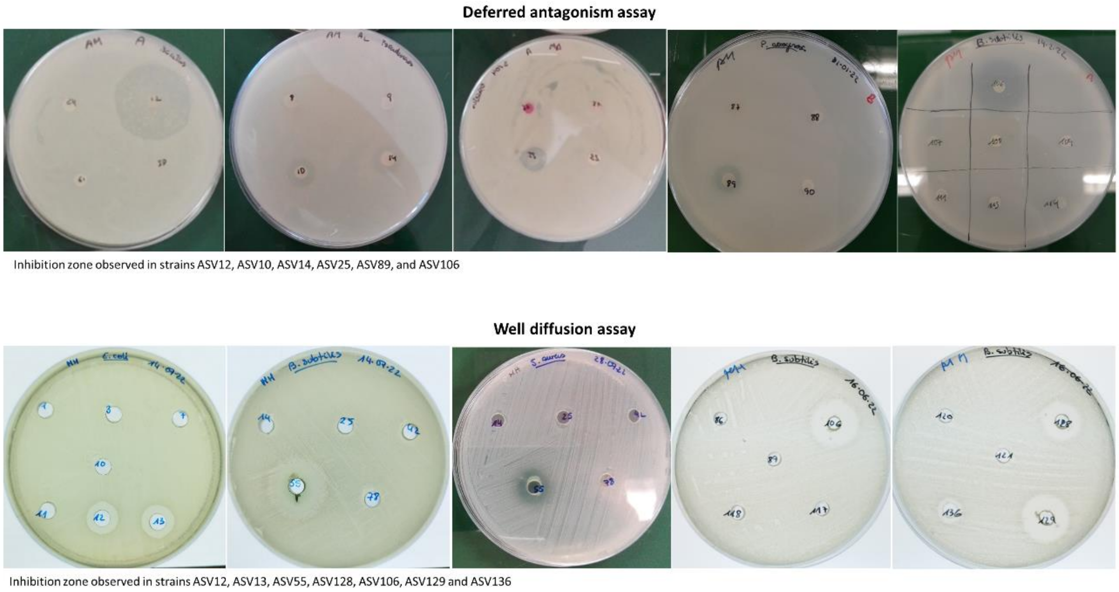

Figure S3. Visual example of some of the strains that produce antimicrobial compounds in different assays.

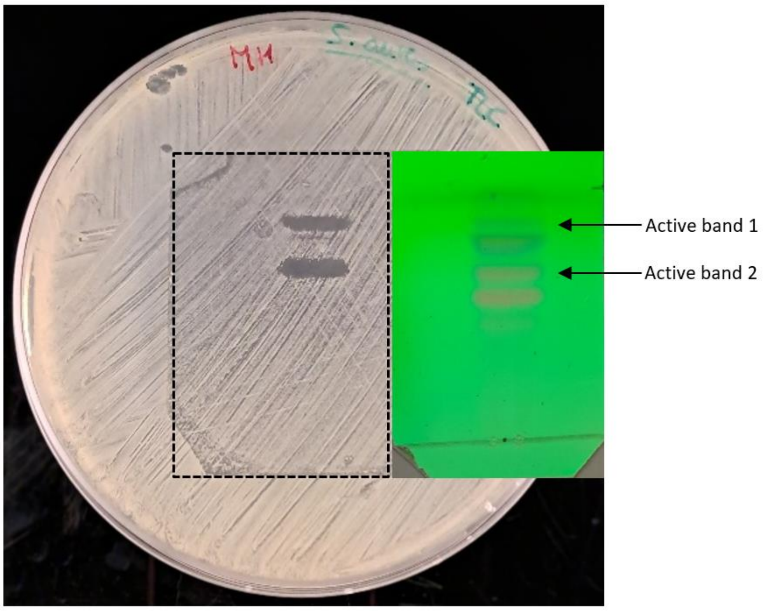

Figure S4. TLC-based bioauthography of crude extract of ASV78 exhibiting antibacterial activity against *S. aureus* ATCC 29213.

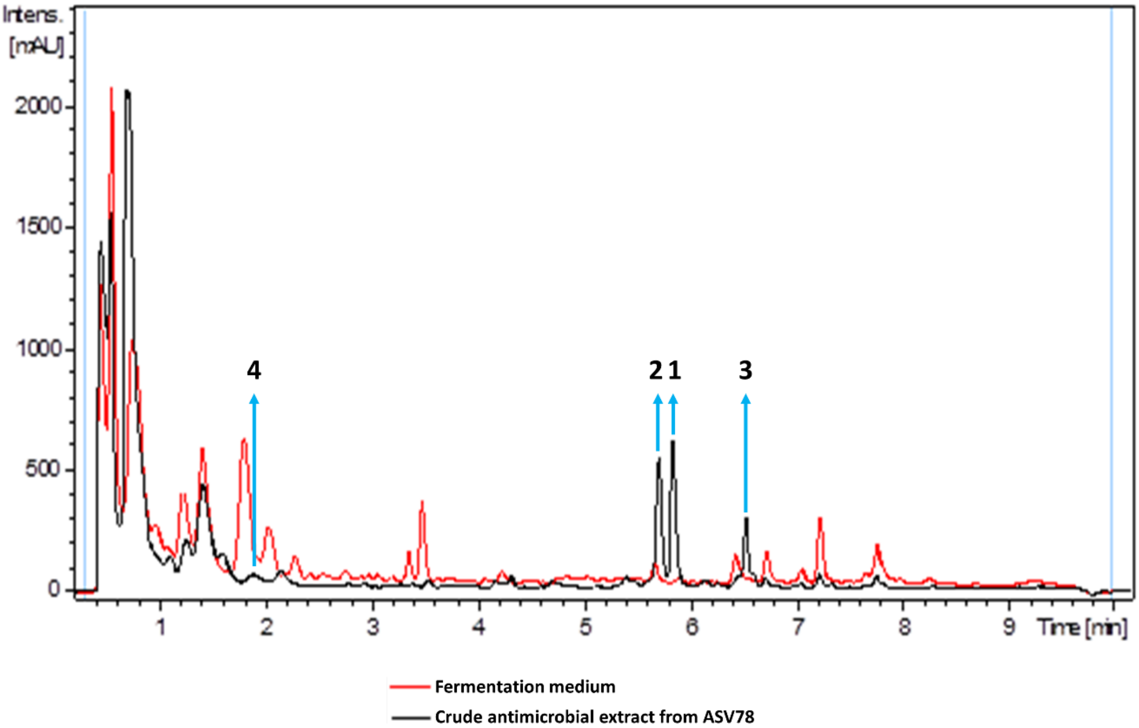

Figure S5. The entire LC-HRMS profile of the crude antimicrobial extract showing the 4 differential peaks with respect to the fermentation medium (without inoculum).
1) pentabromopseudilin; 2) bromophene; 3) molecular formula without coincidences in the Dictionary of Natural Products; 4) lumichrome.

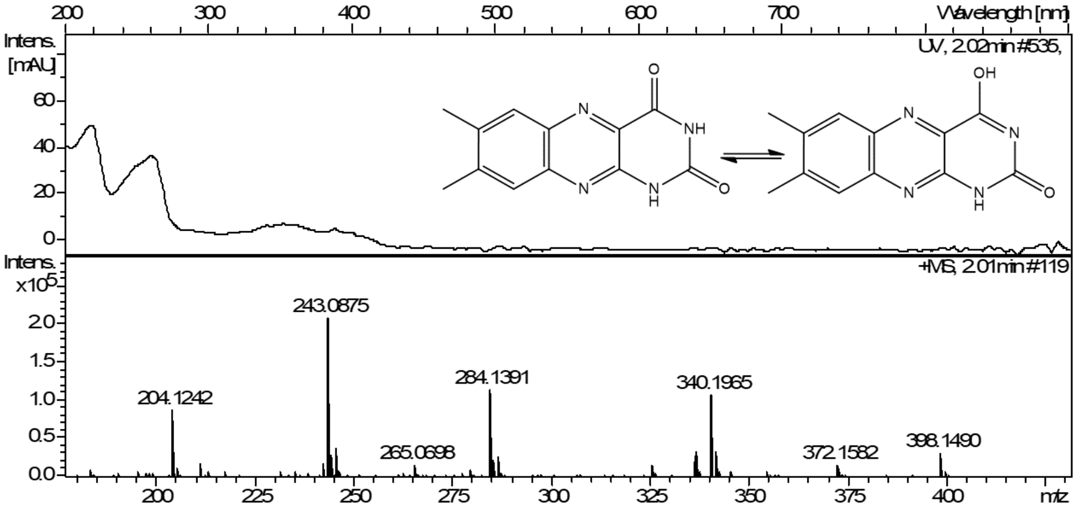

Figure S6. UV and (+)-HRMS spectra of peak dereplicated as lumichrome. Ion at m/z 243.085 was interpreted as [M+H]+ of a compound with a molecular formula $C_{12}H_{10}N_4O_2$. Its UV spectrum and retention time of elution in standardized MEDINA HPLC system were in perfect concordance with lumichrome's analytical data stored in MEDINA's spectral library.

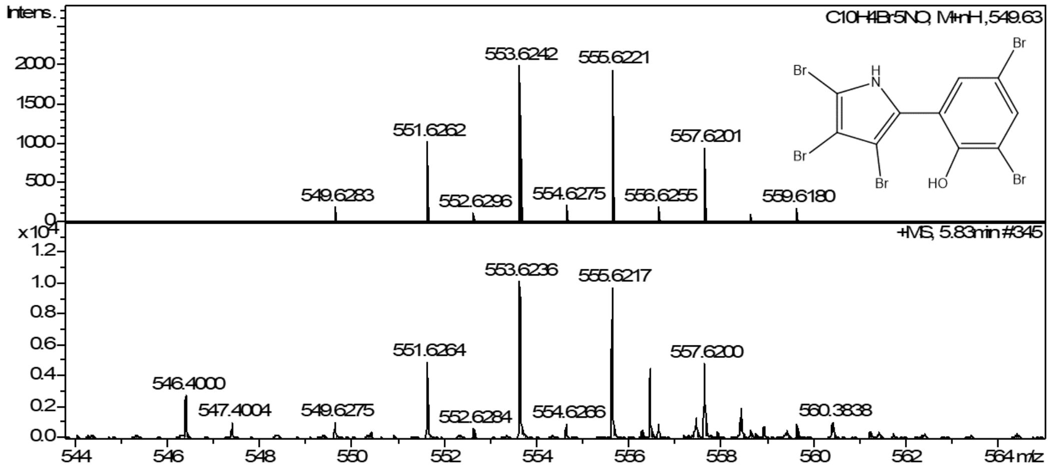

Figure S7. (+)-HRMS spectrum of component dereplicated as pentabromopseudilin (lower) and simulated pattern of $[C_{10}H_5Br_5NO]$+ (upper). Both spectra are perfectly compatible by isotopic pattern and exact masses of ions, allowing the assignment of the interpreted molecular formula to the analyzed peak. Only pentabromopseudilin is described with the molecular formula $C_{10}H_4Br_5NO$ in the Dictionary of Natural Products.

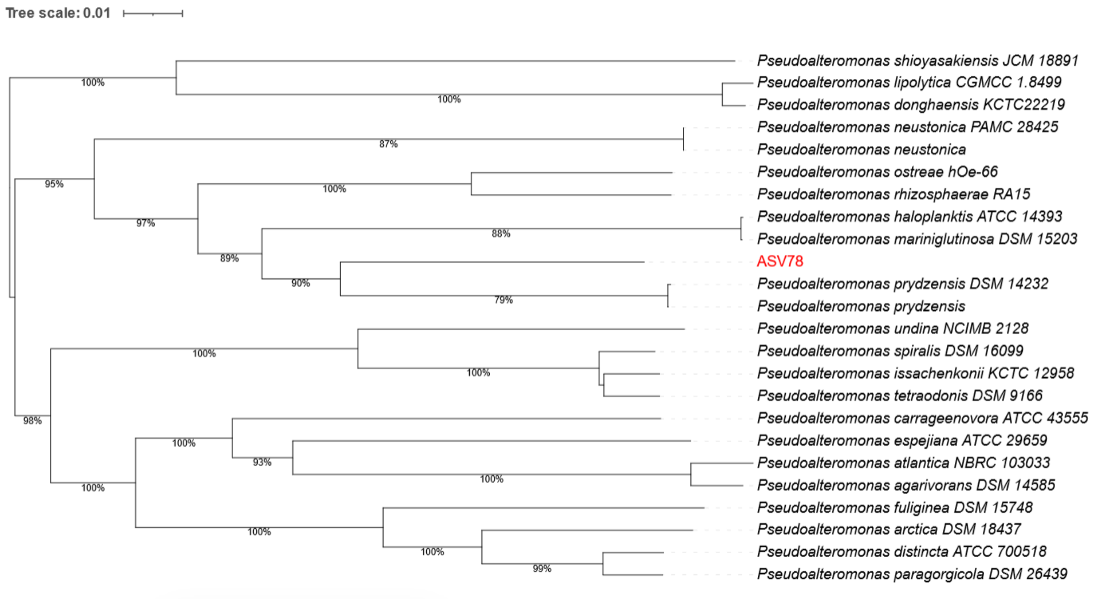

Figure S8. Phylogenomic tree showing the position of the strain ASV78 and related strains of *Pseudoalteromonas* species based on genome distances as provided by the TYGS platform. Bootstrap values greater than 50% in the clade nodes are shown. Bar, 0.01 substitutions per nucleotide position.

TABLES

Table S1. Halophilic and halotolerant isolates obtained from sampling sites. The isolate ID, culture medium from which they were isolated, macroscopic description of colony and cell morphology and Gram staining, and identification based on 16S rRNA gene sequencing (closest species, strains, and sequence similarity) are detailed.

Table S2. The raw data, means and standard deviations of the Parafilm-M test, oil spreading test and emulsification test collected to analyze the production of biosurfactants according to the isolates.

Table S3. Putative biosynthetic gene clusters identified by antiSMASH in the genome of ASV78.

Region	Type	From (bp)	To (bp)	Most similar known cluster (MIBiG Ref)	Similarity
Region 1	PBDE	865,555	889,181	Pentabromopseudilin (BGC0000890)	100%
Region 2	NRPS, Type I PKS	1,224,295	1,296,460		
Region 3	RiPP-like	1,498,707	1,509,561	N-myristoyl-D-asparagine (BGC0000972)	8%
Region 4	NRPS	2,574,952	2,643,824		
Region 5	NI-siderophore	3,032,695	3,063,100	Desferrioxamine E (BGC0001572)	75%
Region 6	Aryl polyene	478,747	522,306	APE Vf (BGC0000837)	45%
Region 7	RiPP-like	804,360	816,540		

PBDE, polybrominated diphenyl ether; NRPS, non-ribosomal peptide synthetase; PKS, polyketide Synthases RiPP, ribosomally synthesised and post-translationally modified peptide product; APE Vf, aryl polyene Vf biosynthetic gene cluster from *Aliivibrio fischeri* ES114.
